# Targeting the Non-catalytic RVxF Site of Protein Phosphatase-1 With Small Molecules for Ebola Virus Inhibition

**DOI:** 10.3389/fmicb.2019.02145

**Published:** 2019-09-13

**Authors:** Xionghao Lin, Tatiana Ammosova, Meng S. Choy, Colette A. Pietzsch, Andrey Ivanov, Asrar Ahmad, Yasemin Saygideğer, Namita Kumari, Dmytro Kovalskyy, Aykut Üren, Wolfgang Peti, Alexander Bukreyev, Sergei Nekhai

**Affiliations:** ^1^Center for Sickle Cell Disease, College of Medicine, Howard University, Washington, DC, United States; ^2^College of Dentistry, Howard University, Washington, DC, United States; ^3^Department of Medicine, College of Medicine, Howard University, Washington, DC, United States; ^4^Yakut Science Centre of Complex Medical Problems, Yakutsk, Russia; ^5^Department of Chemistry and Biochemistry, University of Arizona, Tucson, AZ, United States; ^6^Department of Pathology, Department of Microbiology and Immunology, and Galveston National Laboratory, University of Texas Medical Branch at Galveston, Galveston, TX, United States; ^7^Georgetown Lombardi Comprehensive Cancer Center, Georgetown University, Washington, DC, United States; ^8^Department of Biochemistry, University of Texas Health Science Center, San Antonio, TX, United States

**Keywords:** Ebola virus, protein phosphatase-1, small molecule EBOV inhibitor, protein painting, surface plasmon resonance, split NanoBiT, mass spectrometry

## Abstract

Ebola virus (EBOV) is a non-segmented negative-sense RNA virus that causes a severe human disease. The ongoing EBOV outbreak in the Eastern part of Democratic Republic of the Congo has resulted to date in over 2500 confirmed cases including over 1500 deaths. Difficulties with vaccine administration indicate the necessity for development of new general drugs and therapeutic strategies against EBOV. Host Ser/Thr protein phosphatases, particularly PP1 and PP2A, facilitate EBOV transcription by dephosphorylating the EBOV VP30 protein and switching activity of the polymerase complex toward replication. Previously, we developed small molecule 1E7-03 that targeted host protein phosphatase-1 (PP1) and induces phosphorylation of EBOV VP30 protein thus shifting transcription–replication balance and inhibiting EBOV replication. Here, we developed a new EBOV inhibitor, 1E7-07, that potently inhibits EBOV replication and displays significantly improved metabolic stability when compared to previously described 1E7-03. Proteome analysis of VP30 shows that 1E7-07 increases its phosphorylation on Thr-119 and Ser-124 over 3-fold with *p* < 0.001, which likely contributes to EBOV inhibition. We analyzed 1E7-07 binding to PP1 using a mass spectrometry-based protein painting approach. Combined with computational docking, protein painting shows that 1E7-07 binds to several PP1 sites including the RVxF site, C-terminal groove and NIPP1-helix binding pocket. Further analysis using surface plasmon resonance and a split NanoBiT system demonstrates that 1E7-07 binds primarily to the RVxF site. Together, detailed analysis of 1E7-07 binding to PP1 and identification of the RVxF site as the main binding site opens up an opportunity for future development of PP1-targeting EBOV inhibitors.

## Introduction

Ebola virus (EBOV) is a non-segmented negative-sense RNA virus that causes a severe human disease (Feldmann and Geisbert, 2011). The 2013–2016 epidemic of EBOV in West Africa was the largest in recorded history and resulted in more than 28,000 cases and over 11,000 deaths (Who Ebola Response Team et al., 2016). Moreover, the ongoing EBOV outbreak in the Democratic Republic of the Congo that started in August 2018 has resulted to date in over 2500 confirmed cases including over 1500 deaths (WHO, 2019). Yet, there are no Food and Drug Administration (FDA)-approved drugs for the treatment or prevention of EBOV infection. A cocktail of monoclonal antibodies has been used on individuals infected with EBOV and this treatment is still undergoing clinical trials (The PREVAIL II Writing Group for the Multi-National PREVAIL II Study Team et al., 2016). A recent Merck vaccine showed good protection against EBOV during a phase III trial (Henao-Restrepo et al., 2017), but is not expected to provide protection against additional Ebolaviruses such as Bundibugyo and Sudan, which are equally important human pathogens. Moreover, adverse effects have occurred in about half the people given the vaccine (Henao-Restrepo et al., 2017). Small molecules favipiravir and GS-5734 are also yet to demonstrate success in preventing EBOV infection in their clinical trials (Hayden, 2018). Therefore, it is imperative to develop new general drugs and therapeutic strategies against EBOV.

EBOV encodes several proteins including a nucleoprotein (NP), a polymerase cofactor (VP35), a transcription activator (VP30), a RNA-dependent RNA polymerase (L), a membrane-associated protein (VP24), a matrix protein (VP40) and a glycoprotein (GP) (Nanbo et al., 2013). Previously, we found that the inhibition of the host protein phosphatase-1 (PP1) induces phosphorylation of VP30 and shifts the balance of transcription–replication activity of the polymerase complex toward replication (Ilinykh et al., 2014). We recently extended this study to show that the PP1-targeting compound C31 induces VP30 phosphorylation and inhibits EBOV replication (Ammosova et al., 2018). Recently, NP was shown to recruit the host PP2A-B56 protein phosphatase, which dephosphorylates the VP30 N-terminal serine cluster and upregulates viral transcription suggesting that PP2A also controls VP30 phosphorylation in addition to PP1 (Kruse et al., 2018).

PP1 belongs to the protein phosphatase (PPP) superfamily. The PP1 holoenzyme consists of a catalytic subunit (PP1α, PP1β or PP1γ) and a regulatory, PP1-interacting protein that targets the PP1 holoenzyme into a specific cellular location and determines its activity and substrate specificity (Bollen et al., 2010). Over 200 validated PP1-interactors bind to PP1 catalytic subunits via multiple docking motifs including RVxF, SpiDoc, SILK, MyPhoNE, ΦΦ and NIPP1-helix (Peti et al., 2013; Peti and Page, 2015). Thus small molecules that compete with one or several docking motifs to prevent interactor’s binding to PP1 can be used to functionally disrupt distinct PP1 holoenzymes and inhibit PP1-mediated processes including EBOV replication by changing phosphorylation of viral or host proteins.

Since the RVxF-type docking motif was present in nearly 95% of the validated PP1 interactors (Choy et al., 2014), we attempted to target the RVxF-motif binding pocket with small molecules and identified 1H4 compound (Ammosova et al., 2012). Further modifications yielded PP1-targeting small molecules, 1E7, 1E7-03 and 1E7-04 that were developed as HIV-1 transcription inhibitors (Ammosova et al., 2014). The 1E7-03 showed efficacy against HIV-1 *in vivo* (Lin et al., 2017). Among several analogs tested, 1E7-03 was the least toxic and most potent EBOV inhibitor (Ilinykh et al., 2014). In addition to inhibiting EBOV, 1E7-03 also inhibited Marburg virus (Tigabu et al., 2018), Rift Valley fever virus (Baer et al., 2016), respiratory syncytial virus (Richard et al., 2018), and Venezuelan Equine Encephalitis Virus (Carey et al., 2018). Treatment with 1E7-03 or overexpression of the central domain of the nuclear inhibitor of PP1 (cdNIPP1) induced VP30 phosphorylation and shifted the transcription/replication balance of the EBOV polymerase complex toward replication without cytotoxicity, thereby blocking replication of EBOV (Ilinykh et al., 2014). While 1E7-03 showed good antiviral activity in cultured cells, recent metabolic analysis shows that it is quickly degraded in mice (Lin et al., 2017). 1E7-03 was converted into degradation products 1 (DP1) and DP3 which bound to PP1 *in vitro*, but were not cell-permeable and thus lacked inhibitory activities against HIV-1 (Lin et al., 2017) and EBOV (Ammosova et al., 2018). This instability is likely to impair the *in vivo* antiviral efficacy of 1E7-03. We also recently described an optimized 1E7-03 analog, C31 which binds to the C-terminal groove of PP1 as determined by surface plasmon resonance (SPR) analysis of C31 binding to mutated PP1 (Ammosova et al., 2018). While we showed that 1E7-03 and C31 compete with an RVxF-containing peptide for PP1 binding (Ammosova et al., 2018), the actual binding sites have not been mapped using physical chemistry methods.

In the current study, we optimized the structure of 1E7-03 by developing new analogs and testing them for anti-EBOV activity and metabolic stability. We investigated the effect of the best analog, 1E7-07, on VP30 phosphorylation using label-free quantitative proteomics. Potential PP1 binding sites for 1E7-07 were mapped using a novel protein painting approach, and finally its major binding site was determined using SPR analysis and a (or the) split NanoBiT system (Dixon et al., 2016).

## Materials and Methods

### Chemicals and Reagents

1E7-03 and its five analogs (purity above 98%) were synthesized by Enamine (Kiev, Ukraine) as previously described (Ammosova et al., 2014). Acetonitrile and water containing 0.1% formic acid (FA) were Optima LC/MS grade (Fisher Scientific, Fair Lawn, NJ, United States). High-purity nitrogen (99.9%) was purchased from Roberts Oxygen Co, Inc. (Rockville, MD, United States). Other reagents were of analytical grade. Dimethyl sulphoxide (DMSO), acetone, hydrochloric acid and sodium hydroxide were from Fisher Scientific (Fair Lawn, NJ, United States). Sodium acetate (pH 5.2) was from Quality Biological (Gaithersburg, MD, United States). Phosphate buffered saline (pH 7.4) was from Life Technologies (Grand Island, NY, United States).

### Cells and Media

Vero-E6 and HEK293T cells were purchased from the American Type Culture Collection (Manassas, VA, United States). Vero-E6 cells were cultured in modified Eagle medium (Life Technologies) with 10% fetal bovine serum (FBS) and 1% gentamycin (Life Technologies). The 293T cells were cultured in Dulbecco’s modified Eagle’s medium (Invitrogen) containing 10% FBS and 1% antibiotic solution (penicillin and streptomycin).

### High Throughput Screening Assay

All experiments using infectious EBOV were performed under Biosafety Level 4 (BSL-4) conditions at the Galveston National Laboratory. Vero E6 cells (1 × 10^4^ cells/well) were plated in 96-well plates (black clear bottom, Costar) overnight, and the next day compounds were added at 3, 10, and 30 μM using an epMotion 5075 robot (Eppendorf). One-hour post-treatment, EBOV-eGFP was added at an MOI of 0.3 PFU/cell (40 μl) to media containing compound (60 μl) and left on the cells for the course of the experiment. Three days post-infection the mean fluorescence intensity (MFI) was measured using an EnVision plate reader.

### Mini-Genome System

Minigenome was assembled as previously described (Edwards et al., 2015), using plasmids pCEZ-NP, pCEZ-VP35, pCEZ-VP30, pCEZ-L, and pC-T7 that were kindly provided by Dr. Yoshihiro Kawaoka. Briefly, plasmids pCEZ-NP, 0.25 μg; pCEZ-VP35, 0.25 μg; pCEZ-VP30s, 0.15 μg; pCEZ-L, 2.0 μg; pCT7, 0.5 μg; mono-cistronic minigenome, 0.5 μg were co-transfected in 293T cells using Mirus transfection reagent (Mirus Bio). Six hr post-transfection, the cells were treated with 10 μM 1E7-03 or 10 μM 1E7-07. Transcription was measured at 48 h post-transfection by the luciferase assay (Promega) and normalized to viable cell number.

### EBOV-eGFP Titration

Vero-E6 cell monolayers were grown in 24 well plates and treated with compounds diluted in maintenance MEM medium (Life Technologies, Grand Island, NY, United States) containing 2% FBS (Hyclone, Logan, UT), 0.1% gentamicin sulfate (Corning, Manassas, VA, United States), 1% Non-essential Amino Acids (Sigma, St. Louis, MO, United States), and 1% sodium pyruvate (Sigma) for 1 h at 37°C, 5% CO_2_. Cell monolayers were infected with the recombinant EBOV that expresses eGFP (EBOV-eGFP) from an added gene (Hoenen et al., 2013) for 3 h at 37°C. Following adsorption, monolayers were washed three times with PBS, fresh compound in the maintenance medium was added to each well, and monolayers were incubated for 48 h at 37°C. To determine titers of EBOV-eGFP, supernatants were titrated on Vero-E6 cell monolayers covered with an overlay of the medium containing 0.9% methylcellulose (Sigma). After 4 day-long incubation at 37°C, fluorescent viral plaques were counted under a UV microscope. Experiments with EBOV-eGFP were performed in the BSL-4 facilities of the Galveston National Laboratory, UTMB.

### Cell Viability Assays

To assess cell viability, 1 × 10^4^ cells/well of Vero-E6 cells were plated in 96-well plates (white polystyrene, Costar) overnight and treated with compounds (30, 10, and 3 μM) as above. Cell viability was assessed 5 days after compound treatment using Viral ToxGlo (Promega), and ATP content was determined by reading luminescence using a BioTek Synergy HT plate reader.

293T cells grown in 96-well plates were treated overnight with compounds or DMSO. To assess cytotoxicity with MTT, 10 μl of MTT solution (ATCC^®^ 30-1010K^TM^) was added to each well and incubated under 37°C for 2 h, after which the medium was discarded, and 100 μl of DMSO was added to each well. The cells were incubated for 20 min. The UV absorbance was measured at 490 nm at a microplate spectrophotometer (Bio-Rad Model 680, United States). Each point was triplicated and serum free medium with MTT solution was used as a negative control.

### LC/FT-MS Analysis of Small Molecules

Ten μl aliquots from each sample were loaded on to a LC-20AD nano HPLC system (Shimadzu Corporation, Columbia, MD, United States) coupled to LTQ XL Orbitrap mass spectrometer (Thermo Fisher Scientific) with the installed Xcalibur software (version 2.0.7, Thermo Fisher Scientific). Liquid chromatography was carried out on an in-house made nano-HPLC column (Polymicro Technologies Inc., Phoenix, AZ, United States) packed with reverse phase PolySulfoethyl A, 5 μM, 200 Å (PolyLC Inc., Columbia, MD, United States). Mobile phase A was 0.1% formic acid in water and mobile phase B was 0.1% formic acid in acetonitrile. The elution was performed at a flow rate of 600 nl/min over 40 min using a multi-segment linear gradient of mobile phase B as follows: 0–6.02 min, 1% B; 6.02–6.11 min, 1–2% B; 6.11–20 min, 2-80% B; 20–25 min, 80% B; 25–30 min, 80–85% B; 30–31 min, 80–2% B; 31–40 min, 2% B (v/v). The Orbitrap was operated under data-dependent acquisition mode. The spray voltage, capillary temperature and capillary voltage were set to 2.0 kV, 200°C, and 39.5 V, respectively. Full-scan mass spectra were acquired in Orbitrap over 150-1500 *m/z* with a resolution of 30,000, followed by MS^n^ scans by collision-induced dissociation (CID) activation mode. The precursor ions were fragmented with collision energy of 18 with activation Q of 0.25 and an activation time of 30 ms. Dynamic exclusion was enabled with a repeat count of 2, a repeat duration of 15 s, an exclusion duration of 20 s, an exclusion list size of 50.

### Stability of Compounds in Human and Mouse Serum

1E7-07 or 1E7-03 dissolved in DMSO (10 mM stock solution) was mixed with human or mouse serum (H3667, M5905, Sigma-Aldrich) to a final concentration of 10 μM and incubated at 37°C. Aliquots were collected at different time points for up to 24 h. The resulting sample (50 μl) was mixed with 200 μl of cold acetone, vortexed for 2 min, kept at −20°C for 30 min, and then the precipitated protein was removed by centrifugation at 13,000 × *g* for 5 min. The supernatant was transferred to a clean test tube and evaporated to dryness using a SpeedVac concentrator. The residue was reconstituted in 50 μl of acetonitrile for LC/FT-MS analysis. All experiments were run parallel in triplicates.

### Stability of Compounds in Liver Microsomes

The metabolic stability of compounds was determined by using a pool of human and mouse liver microsomes (HMS9PL, MSS9PL, Thermo Scientific) following manufacturer’s instructions. In brief, incubations were conducted at 37 ± 1°C in mixtures containing 5 μl of human or mouse liver microsomes (20 mg/ml), 183 μl of potassium phosphate buffer (pH 7.4, 100 mM) and 2 μl of 1E7-07 or 1E7-03 (10 mM sock solution of each compound in DMSO was diluted to 1 mM with PBS). The mixture was pre-incubated for 5 min, then the reactions were initiated with the addition of 10 μl 20 mM NADPH (Sigma Aldrich). The reactions were terminated after 1 h incubation by adding 800 μl cold acetone. The mixture was kept at 4°C for 30 min, and the precipitated protein was removed by centrifugation (13,000 *g* for 10 min at 4°C). A 250 μl aliquot of supernatant was transferred to a clean test tube and evaporated to dryness using a SpeedVac concentrator. The residue was reconstituted in 50 μl of acetonitrile with 0.1% FA for LC/FT-MS analysis.

### Stability of Compounds in Cell Culture Media

293T cells seeded in 24-well plates (2 × 10^5^ cells/well) were treated the next day with 10 μM of 1E7-07 or 1E7-03. Media (100 μl) was collected at different time points during 48 h and total protein was precipitated by 400 μl of cold acetone and centrifuged at 13,000 × *g* for 5 min. The supernatant was collected and evaporated to dryness using a SpeedVac concentrator. The dry pellet was reconstituted in 100 μl of acetonitrile for LC/FT-MS analysis.

### Degradation of Compounds in Solutions With Different pH

1E7-07 or 1E7-03 (10 μM) stability at various pH was tested at 37°C in sodium acetate-acetic acid buffer (pH = 4), NaH_2_PO_4_/Na_2_HPO_4_ buffer (pH = 7) and NaHCO_3_/NaOH buffer (pH = 10). Samples (100 μl) were collected at different time points up to 48 h, and evaporated to dryness using a SpeedVac concentrator immediately. The pellets were resolved in 100 μl of acetonitrile, vortexed for 2 min, then centrifuged at 13,000 × *g* for 5 min. The supernatant was transferred to a clean tube for LC-MS analysis.

### Dephosphorylation Assay

Malachite green dephosphorylation assays were carried out with the Ser/Thr phosphatase assay kit (Upstate, Lake Placid, NY, United States) using recombinant PP1α (New England Biolabs, Ipswich, MA, United States) as described previously (Ammosova et al., 2012). About 0.005U of PP1α was incubated with KT(pT)IRR peptide (Upstate, Lake Placid, NY, United States). The reactions were carried out in PP1 reaction buffer (50 mM Tris-HCl pH 7.5, 100 mM NaCl, 2 mM dithiothreitol, 0.1 mM EGTA, 0.025% Tween-20) supplemented with 1 mM MnCl_2_ (New England Biolabs) in 25 μl reaction volume with the indicated concentrations of 1E7-07 or 1E7-03. After reaction, 25 μl aliquots were removed and mixed with 100 μl of Malachite Green solution (Upstate). Absorbance of malachite green was determined at 620 nm and the phosphate concentration was recalculated using a calibration curve of phosphate standards prepared using 1 mM KH_2_PO_4_ solution.

### Label-Free Quantitative Analysis of VP30 Phosphorylation

HEK293T cells (40% confluence) were transfected with Flag-VP30 expression vector, using Lipofectamine 3000/Plus in OPTI-MEM as directed by the manufacturer. Twenty four h post-transfection, the cells were treated overnight with 10 μM of 1E7-07 or DMSO as a control. VP30-transfected cells were also treated with 100 nM okadaic acid for 2 h as an additional control. VP30 was immunoprecipitated from cellular lysates with anti-Flag antibody, resolved by 10% sodium dodecyl sulfate–polyacrylamide gel electrophoresis (SDS-PAGE), digested in gel and submitted for LC/FT-MS analysis.

A total of 10 μl of sample was loaded and washed for 6 min on a C_18_-packed precolumn (1 cm × 150 μm, 5 μm, 200 Å, Michrom Bioresources, Auburn, CA, United States) with a solvent of A:B = 99:1 (A, 0.1% formic acid aqueous solution; B, 0.1% formic acid acetonitrile solution) at a constant flow of 12 μl/min. Peptides were transferred onward to an in-house C_18_-packed analytical column (25 cm × 150 μm, 5 μm, 200 Å, Michrom Bioresources, Auburn, CA, United States) and separated with a linear gradient of 6–55 min, 2–40% B, 55–62 min, 40–80% B, 62–70 min, 80% B (v/v) at a flow rate of 600 nl/min. Mobile phase A was 0.1% formic acid in water and mobile phase B was 0.1% formic acid in acetonitrile. The Orbitrap was operated under data-dependent acquisition mode. The spray voltage, capillary temperature and capillary voltage were set to 2.0 kV, 200°C, and 39.5 V, respectively. Full-scan mass spectra were acquired in Orbitrap over 300–2000 *m/z* with a resolution of 30,000, followed by MS^n^ scans by CID activation mode. The three most intense ions were selected for fragmentation using CID in the LTQ (normalized collision energy of 35, parent mass selection window of 2.5 Da, activation time of 30 ms, and minimum signal threshold for MS/MS scans set to 500 counts). Charge state rejection (charge state 1 was rejected) as well as dynamic exclusion (repeat counts, 2; repeat duration, 10 s; exclusion duration, 10 s) was enabled.

LC-MS/MS raw data were searched using Proteome Discoverer 1.4 (PD 1.4) with the SEQUEST search engine (Thermo Fisher Scientific), against the Flag-VP30 added EBOV protein database (11/29/2017, 140705 sequences). The FASTA database was concatenated with proteins of African green monkey and common contaminants. A maximum of two missed cleavage sites were allowed. The mass tolerance for the precursor ion was set to 30 ppm and for the fragment to 0.1 Da. Phosphorylation of serine and threonine were enabled as dynamic modifications, while carbamidomethylation of cysteine was set as fixed modification. Filter settings for peptides with different charges are: charge 2 = 1.5, charge 3 = 2.0, and charges > 4 = 2.5 for high confidence peptides; charge 2 = 0.5, charge 3 = 0.8, and charges > 4 = 1 for modest confidence peptides. The label-free quantification of phosphopeptides eluting between 10 and 70 min was performed with SIEVE 2.1 software (Thermo Scientific). Briefly, the chromatographic peaks detected by Orbitrap were aligned and the peptide peaks were detected with a minimum signal intensity of 1 × 10^5^; quantitative frames were determined based on *m/z* (width: 10 ppm) and retention time (width: 2.5 min). The identified VP30 peptides by PD 1.4 were unloaded as framing seeds. Statistical filters were set to assess the quality of the data. The CV raw MS intensities of the triplicates had to be within 25%. This helped minimize the effect of run-to-run variability.

### Molecular Painting

Three groups (i, ii, and iii) of molecular painting reactions were set up. In each group, recombinant protein PP1 was dissolved in 300 μl of a labeling solution consisting of solution A (PBS) and solution B (0.2 M sodium bicarbonate solution was adjusted to pH 9.0 with 2 M sodium hydroxide) with a ratio (20:1, A:B) at a final concentration of 10 nM. Group iii was mixed with 6 μl of 10 mM 1E7-07 in DMSO at room temperature for 30 min, while groups i and ii were mixed with the same volume of DMSO. Next, 20 μl of ELNLLB (EZ-Link NHS-LC-LC-Biotin, 21343, Thermo Scientific) was added into groups ii and iii and incubated for another 30 min, and DMSO was added into group i as control. After the reaction was complete, compound 1E7-07 and painting molecule ELNLLB were removed by centrifugal filters (OD003C34, Life Sciences) at 1,2000 rpm for 2 min with five times wash of 200 mM Ammonium Bicarbonate. The solutions on the top of the filters were collected and adjusted to 200 μl. All samples were denatured and reduced in 10 mM dithiothreitol (1 h at 37°C), alkylated with 30 mM iodoacetamide (20 min, room temperature in the dark) and digested for 3 hr with 2 μg trypsin (Promega) at 37°C on a dry bath. Tryptic peptides were purified by Zip-Tip (Millipore) following manufacturer’s instructions, and analyzed by reversed-phase liquid chromatography nanospray tandem MS (LC-MS/MS) using an LTQ Orbitrap mass spectrometer (Thermo Fisher). Label-free quantification of peptides derived from PP1 was performed with SIEVE 2.1 software as above. The identified peptides of PP1 by PD 1.4 were unloaded as framing seeds.

### Molecular Docking

The Blind Docking Web Server^1^ was used for docking simulations. The tool performs a series of docking calculations across the protein surface in order to find the spots with best binding affinities. After the affinities are calculated, the tool clusters the results according to spatial overlapping of the resulting poses. For each cluster, the pose with the best affinity is taken as the representation. Figures were prepared using the PyMOL 1.5.

### PP1α Expression and Purification

Recombinant PP1α was prepared as previously described (Ammosova et al., 2014). BL21 (DE3) *Escherichia coli* cells (Invitrogen, Carlsbad, CA, United States) were co-transformed with a vector RP1B-PP1α7-300, which expresses human PP1α (residues 7–300) and pGR07 (Takara), which expresses GroEL/GroES chaperones (both gifts from Dr. Mathieu Bollen and Monique Beullens, KU Leuven, Belgium). The cells were grown in media supplemented with 1 mM MnCl_2_ at 30°C to an A600 ∼0.5. Then arabinose (2 g L^–1^) was added to induce expression of the GroEL/GroES chaperones. When A600 ∼1 was reached, the cells were transferred to 10°C and PP1α expression was induced with 0.1 mM Isopropyl β-D-1-thiogalactopyranoside for 20 h. Harvested cells were lysed using high pressure homogenizer Avestin Emulsiflex C3 in a solution containing in 50 mM Tris-HCl (pH 8.0), 5 mM imidazole, 700 mM NaCl, 1 mM MnCl_2_, 0.1% Triton X-100 (v/v) and protease inhibitors. His-tagged PP1 was purified using a Ni-NTA IMAC column (Qiagen, Valencia, CA, United States). PP1 was further purified using size exclusion chromatography in SEC buffer (20 mM Tris pH 8.0, 500 mM NaCl, 0.5 mM TCEP, 1 mM MnCl_2_). Glycerol (50% v/v) was added to the purified PP1 and the PP1 was aliquoted, flash frozen and stored at −80°C.

### Surface Plasmon Resonance (SPR)

Surface plasmon resonance measurements were conducted on the Biacore T200 instrument (GE Healthcare, Piscataway, NJ, United States) at 25°C. Recombinant PP1 was immobilized on a CM5 chip by amine coupling (GE Healthcare). PP1 (200 nM) was captured on flow cell 2 in 10 mM acetate buffer, pH 5.0, supplemented with 2 mM MnCl_2_. The average amount of PP1 immobilized on the surface was 3160 RU. Flow cell 1 was used as a reference surface to subtract background signal. Injections of the buffer alone were used to provide double reference subtraction. To measure the direct binding of small molecules to PP1, the two flow cells of the sensor chip were primed with running buffer (0.01 M HEPES pH 7.4, 0.15 M NaCl, 0.005% v/v Surfactant P20, 1% DMSO and 2 mM MnCl_2_). For the competition assay, the hybrid peptide consisting of parts of the pRb and Tat protein sequences, HIPR(pS)PYKFPSSPLRKKCCFHCQVCFITK (with a single serine amino acid phosphorylated) was used at 10, 25, 50, 75, and 100 nM. Compound 1E7-07 was used at 1 μM. In contrast, pRb-Tat peptide was used at 25 nM, whereas 1E7-07 was used at 0.625, 1.25, and 2.5 μM. For binding and kinetics experiments of 1E7-07 on WT PP1 and MUT PP1, compound was diluted in the running buffer, at 40, 20, 10, 5, 2.5, 1.25, and 0 μM and then passed over the two flow cells at a flow rate of 100 μl/min for 60 s. The number of response units was recorded after the subtraction of the reference flow cell’s value (Fc2-1). Two repetitions were performed for each injection. Data were analyzed using the BiaEvaluation software of Biacore with a 1:1 binding model using Global fitting.

### Plasmids and Constructs

Plasmids including the human PP1, PP1 MUT (Y70W, L73Y, G274E, and A299E), cdNIPP1 (Residues 140-225), cdNIPP1 RATA (V201A and F203A) and cdNIPP1 helix MUT (D164A, T171A, N174A, K175A, and I177A) coding sequences were obtained from Addgene. Genes of interest were subcloned into pFC34K vector to generate the C-terminal LgBiT-fused PP1/PP1 MUT and C-terminal SmBiT-fused cdNIPP1/cdNIPP1 RATA/cdNIPP1 helix MUT using *Sgfl* plus *Pmel* restriction sites.

PP1γ-mCherry expression vector was constructed as follows. pcDNA3.1(-) plasmid was digested with Not1 and Kpn1 restriction enzymes and purified on the agarose gel. The mCherry fragment was amplified by PCR from pcDNA3.1-mCherry plasmid with forward GATCACAAAGCAAGCAAAGAAAAAGGGCGAGGAGGATA ACATGG and reverse GTTTAAACTTAAGCTTGGTACCTTAC TTGTACAGCTCGTCCATGCC primers. PCR fragment of PP1γ was amplified with forward GATCACAAAGCAAGCA AAGAAAAAGGGCGAGGAGGATAACATGG and reverse GT TTAAACTTAAGCTTGGTACCTTACTTGTACAGCTCGTCCA TGCC primers from PP1γ-sds22 expression vector provided by Dr. Mathieu Bollen. All fragment were incubated in Gibson assembly kit (New England BioLab E2611S) according to the standard protocol.

### NanoBiT Assay

HEK293T cells were plated in 96-well white/clear culture plates with 40% confluence and transiently transfected with the indicated constructs (1:1 ratio of interacting pairs) using Lipofectamine 3000 Plus in OPTI-MEM as directed by the manufacturer. Twenty-four h post transfection, cells were treated with serial concentrations (1.3–14 μM) of 1E7-07 for an additional 6 h. Nano-Glo Live Cell Substrate (N2012, Promega) was added and luminescence was measured using a GloMax-Multi Detection System (Promega).

### Fluorescent Microscopy

To analyze the effect of 1E7-07 on PP1, 293T cells were transfected with vectors expressing PP1γ-mCherry or a combination of PP1γ-mCherry and cdNIPP1-EGFP (Ammosova et al., 2005). At 24 h post transfection, the cells were treated with 1E7-07 or DMSO as vehicle control. At 48 h posttransfection the cells were photographed on Olympus IX73 using filters for Texas Red and FITC fluorescence with 600X or 400X magnification.

### Statistical Analysis

All graphs were prepared using GraphPad prism 6 software. Data are presented as mean ± SD or standard error of the mean (SEM) as indicated in the figure legends. Means were compared with Student *t*-tests.

## Results

### Structural Optimization of 1E7-03

**FIGURE 1:**
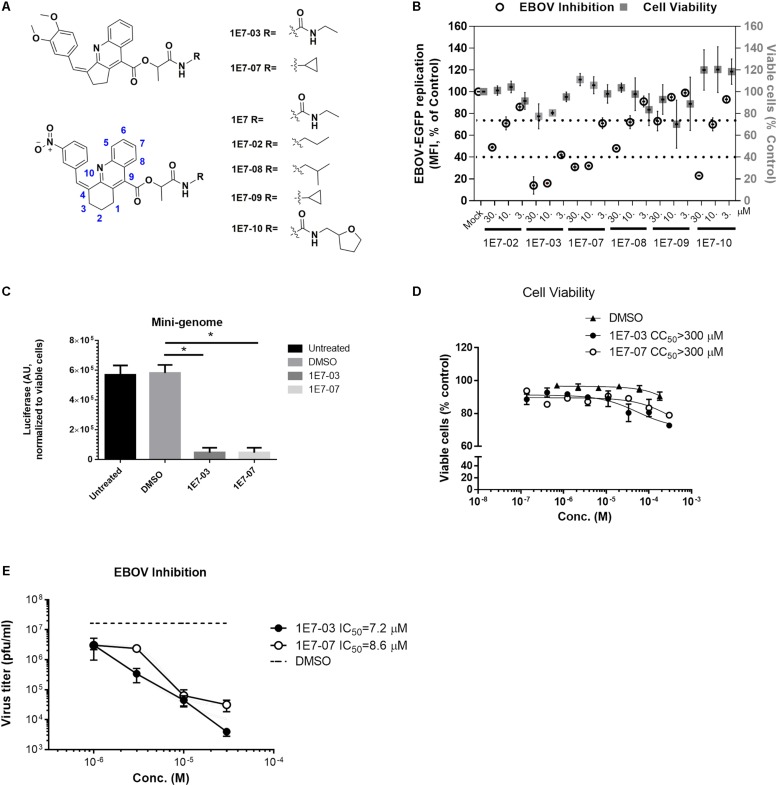
Development of EBOV inhibitory 1E7-07 compound with optimized metabolic stability. **(A)** Chemical structures of 1E7-03 analogs synthesized by Enamine. **(B)** EBOV inhibition and cytotoxicity of 1E7-03 analogs. To measure the antiviral activity of the compounds, Vero-E6 cells were plated in 96-well plates overnight and then pre-treated with increasing concentrations of compound for 1 h, after which they were infected with EBOV-eGFP (MOI = 0.3). Three days post-infection the mean fluorescence intensity (MFI) of expressed eGFP was determined and normalized to untreated controls to determine percent infection. In addition, the cell viability was measured using Viral ToxGlo assay. Data are the mean ± SD of triplicates. **(C)** EBOV inhibition by 1E7-07 was measured by minigenome assay. HEK293T cells were transfected with the components of the EBOV minigenome system in 96-well plates. Six hour post-transfection, cells were treated with 1E7-07, 1E7-03, or DMSO. Forty-eight hour post-transfection luciferase activity was measured. Data are the mean ± SD of triplicates. ^∗^*p* < 0.001, 1E7-03 or 1E7-07 treated group versus DMSO group. **(D)** Cytotoxicity of 1E7-07 compared with 1E7-03 in 293T cells analyzed by MTT assay. Each point was run in triplicate and serum free medium with MTT solution was used as a negative control. **(E)** EBOV inhibition by 1E7-07. Triplicate Vero-E6 cell monolayers were treated with the indicated concentrations of 1E7-07 or 1E7-03, then infected with EBOV-eGFP and supernatants were collected at 48 h post infection. Virus titer was measured, and EBOV-eGFP titers ± SD are shown.

We recently showed that 1E7-03, a PP1-targeting compound was degraded *in vivo* (Lin et al., 2017). Its degradation products were not cell permeable, and thus lacked EBOV inhibitory activity (Ammosova et al., 2018). To improve the metabolic stability of 1E7-03, we designed several analogs (Figure 1A) based on the 1E7 lead compound that was initially used to develop 1E7-03 (Ammosova et al., 2014). The lead 1E7 compound (reported as T5236177 or compound 63) inhibited EBOV but was found to be toxic and excluded from further evaluation (Ammosova et al., 2018). The 1E7-based library was designed based on the following criteria (i) position 4 in tetrahydroacridine ring must tolerate small aromatic groups; (ii) the saturated ring in the core must be unsubstituted; and (iii) only flexible linear linkers enriched with hydrogen bonding functionalities are acceptable at position 9 (see more details in Ammosova et al., 2014). Because we observed strong influence of various ethenylbenzene moiety substitutions on anti-HIV-1 activity of 1E7-based compounds (Ammosova et al., 2014), we introduced electron donating groups (OCH_3_, Figure 1A, compounds 1E7-03 and 1E7-07) or electron withdrawing group (NO_2_, Figure 1A, compounds 1E7-02, 1E7-08, 1E7-09 and 1E7-10) and compared their antiviral activities. The analogs were tested for EBOV inhibition with infectious EBOV expressing eGFP (EBOV-eGFP) (Towner et al., 2005). Vero-E6 cell monolayers were pre-treated with the compounds at 3, 10, and 30 μM concentrations for 1 h at 37°C. Following the pretreatment, EBOV-eGFP was added at a multiplicity of infection (MOI) of 0.3 PFU/cell, and viral replication was measured by eGFP fluorescence at day 3 post-infection (Edwards et al., 2015). In parallel, cytotoxicity was determined with Viral ToxGlo (VTG) (Edwards et al., 2015) and MTT assays. Among the tested analogs, 1E7-07 demonstrated the best EBOV inhibitory activity (about 70% at 10 μM) and displayed no toxicity compared to 1E7-03 (Figure 1B). Structurally, 1E7-03 derivatives with *meta*- and *para*- substitution of the methoxy group on the ethenylbenzene moiety achieved better EBOV inhibitory activity than the compounds with *meta*- substitution of the nitro group (Figures 1A,B).

We next analyzed the effect of 1E7-07 on EBOV gene expression using an EBOV minigenome system. HEK293T cells were grown in 96-well white plates and transfected with the EBOV minigenome that expresses a Renilla luciferase reporter (eMGLuc) along with plasmids expressing components of the EBOV polymerase complex (L, NP, VP35, and VP30) under the control of T7 polymerase and a T7 polymerase expressing vector (Ilinykh et al., 2014). Compounds were added at a final concentration of 10 μM and tested in triplicate. Renilla luciferase activity was assessed after 24 h treatment. Compared to the untreated control or vehicle (DMSO) treated control, both 1E7-07 and 1E7-03 strongly inhibit EBOV gene expression (*p* < 0.001), with 1E7-07 showing EBOV gene expression inhibition comparable to 1E7-03 (Figure 1C). To further confirm that 1E7-07 is not toxic, HEK293T cells were treated with 1E7-07 and 1E7-03 compounds at different concentrations (0.1–300 μM), and cellular viability was analyzed by MTT after 24 h incubation. Both 1E7-07 and 1E7-03 had a CC_50_ over 300 μM and had no effect on cellular viability at concentrations below 30 μM (Figure 1D). To test the effect of 1E7-07 on the production of EBOV viral particles, Vero-E6 cell monolayers were pre-treated with 1E7-07 or 1E7-03 (1–30 μM) for 1 h at 37°C. Then, the cells were infected with EBOV-eGFP (MOI = 2 PFU/cell) for 3 h, followed by three consecutive washes with sterile PBS and replacement with media containing test compounds. After 48 h incubation, supernatants were collected and viral titers were determined. Treatment with 1E7-07 and 1E7-03 resulted in a similar dose-dependent inhibition of virus replication with IC_50_ = 8.6 μM for 1E7-07 and IC_50_ = 7.2 μM for 1E7-03 (Figure 1E).

### 1E7-07 Demonstrates Improved Metabolic Stability Over 1E7-03

**FIGURE 2:**
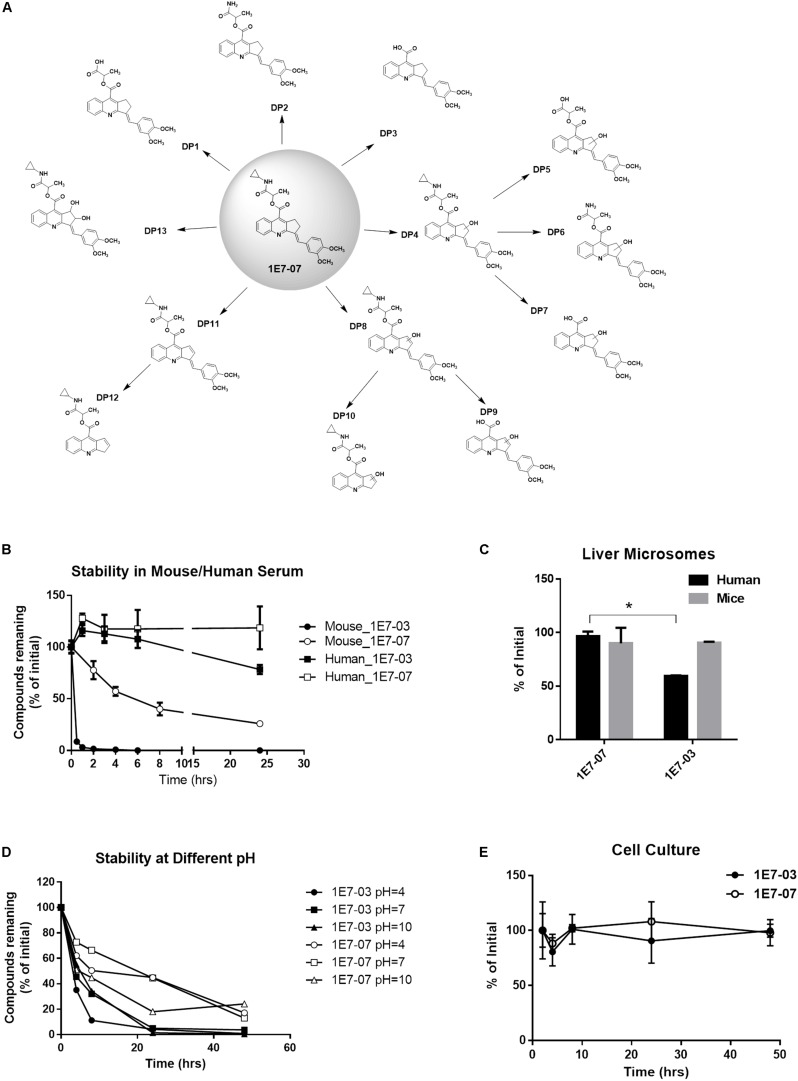
Cytotoxicity and stability of 1E7-07. **(A)** Degradation pattern of 1E7-07. 1E7-07 was incubated under different experimental conditions, including mouse plasma (24 h) and buffers with pH 4, pH 7, and pH 10 (48 h) at 37°C. Degradation products (DPs) were extracted with cold acetone precipitation and identified by LC/FT-MS analysis. A total of 13 DPs were identified. **(B)** Stability of 1E7-07 in human and mouse serum. 1E7-07 or 1E7-03 (10 μM) was added to human or mouse serum and incubated at 37°C for 24 h. Samples were collected at different time points, and compounds were extracted and quantified by LC/FT-MS analysis. **(C)** Stability of 1E7-07 in liver microsomes from human and mouse. 1E7-07 or 1E7-03 dissolved in DMSO (10 mM stock solution) was mixed with liver microsomes to the final concentration of 10 μM and incubated at 37°C for 1 h. Samples were processed and analyzed by LC/FT-MS. Shown are the means ± SD of triplicates. ^∗^*p* < 0.001, 1E7-07 versus 1E7-03 in human liver microsomes. **(D)** Degradation kinetic of 1E7-07 under different pH. 1E7-07 or 1E7-03 (10 μM) was incubated at 37°C in sodium acetate-acetic acid buffer (pH = 4), NaH_2_PO_4_/Na_2_HPO_4_ buffer (pH = 7) and NaHCO_3_/NaOH buffer (pH = 10). Samples collected at different time points up to 48 h and processed for LC-MS analysis. **(E)** Stability of 1E7-07 during the cell culture. 1E7-07 or 1E7-03 (10 μM) was added to the media and incubated at 37°C for 48 h. Samples were collected at different time points. Remaining compounds were extracted and quantified by LC/FT-MS analysis.

We next compared stability of 1E7-07 to 1E7-03 in human and mouse serum at 37°C during 24 h incubation. Compounds (10 μM) were added to serum and aliquots were collected at different time points. Compounds were extracted from serum after total protein was precipitated with acetone, and quantified using LC/FT-MS (Lin et al., 2017). We identified 1E7-07 degradation products that were observed during incubation in mouse serum and in buffers with pH 4, pH 7, and pH 10 for 48 h at 37°C (Figure 2A). The degradation patter was similar to 1E7-03 (Lin et al., 2017) except that 1E7-07 was more stable in mouse serum (Figure 2B). 1E7-03 was degraded by 90% in less than 30 min and by 100% in 6 h. In contrast, 1E7-07 had a half-life of 6 h (Figure 2B) and 25% 1E7-07 remained in serum after 24 h of incubation (Figure 2B). Both 1E7-03 and 1E7-07 showed better stability in human serum than in mouse serum with 78% remaining for 1E7-03 and no degradation for 1E7-07 after 24 h incubation (Figure 2B). Next, we tested metabolic stability of 1E7-07 in human and mouse liver microsomes (Figure 2C). In mouse liver microsomes both compounds were stable and over 90% of 1E7-07 and 1E7-03 remained intact after 1 h incubation (Figure 2C). However, 1E7-07 showed better stability in human liver microsomes compared to 1E7-03 (90% vs. 60% remaining, *p* < 0.001, Figure 2C). We also analyzed stability of both compounds at different pH (pH = 4, 7 and 10), and observed overall better stability of 1E7-07 compared to 1E7-03 under all tested conditions (Figure 2D). In addition, we examined 1E7-07 stability in cell culture media. Similarly to 1E7-03, 1E7-07 was stable in complete media during 48 h incubation (Figure 2E).

Taken together, 1E7-07 displayed better overall stability, which could be due to cyclopropyl replacement of the *N*-ethylformamide group in 1E7-03. This replacement may protect the vulnerable amide bond of 1E7-03 which was identified as a hot spot for degradation (Lin et al., 2017). As 1E7-07 shows potent EBOV inhibition activity, we chose this compound for future analysis.

### Effect of 1E7-07 on VP30 Phosphorylation

**FIGURE 3:**
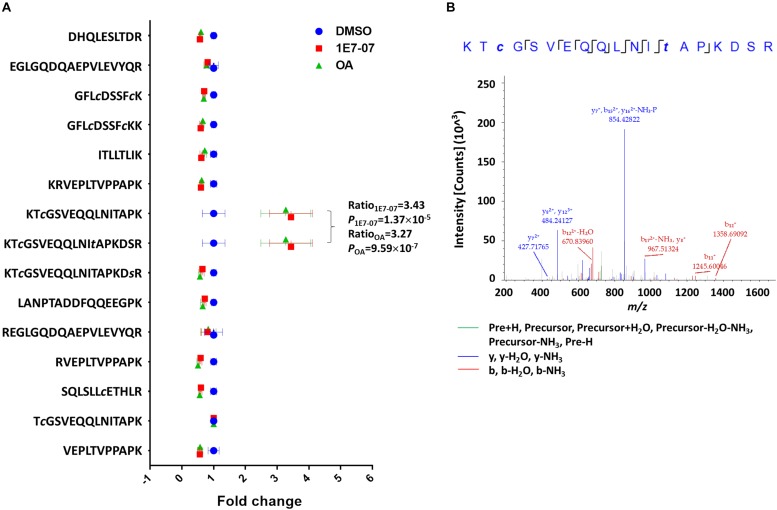
Effect of 1E7-07 on VP30 phosphorylation. **(A)** Label-free quantitative analysis of VP30. HEK293T cells were transfected with Flag-VP30 and treated with 1E7-07 and OA. VP30 was immunoprecipitated from cellular lysates with anti-Flag antibody, resolved by SDS-PAGE, and analyzed by LC-MS/MS. VP30-derived peptides were identified by Proteome Discoverer 1.4. Quantification was performed on SIEVE 2.1 software. Mean intensities (± SD) of the indicated VP30 peptides are shown. Ratio and *P*-value of phosphopeptides KT*c*GSVEQQLNI*t*APKDSR and KT*c*GSVEQQLNITAPKD*s*R were labeled. **(B)** Representative MS/MS spectrum of the phosphopeptide ^107^KT*c*GSVEQQLNI*t*APKDSR^125^. The colored peaks indicate matched MS/MS fragments. Green color indicates precursors, as outlined in the figure; blue and red colors indicate y and b ions, respectively.

We previously demonstrated that 1E7-03 induces phosphorylation of EBOV VP30 (Ilinykh et al., 2014). Another of our recent studies showed that PP1-targeting C31 compound induces VP30 phosphorylation (Ammosova et al., 2018). Thus, we tested the effect of 1E7-07 on VP30 phosphorylation in cultured cells and compared it to okadaic acid. Flag-tagged VP30 was expressed in HEK293T cells which were treated with 10 μM 1E7-07 overnight, 0.1 μM okadaic acid for 2 h or DMSO as vehicle control. VP30 was immunoprecipitated from cellular lysates with anti-Flag antibody, resolved on 10% SDS-PAGE, and analyzed by LC-MS/MS. Proteins were identified with Proteome Discoverer 1.4 followed by label-free quantification with SIEVE 2.1 software. Fifteen peptides derived from Flag-VP30 were quantified with a relative standard deviation (RSD) < 25% (Figure 3A). Phosphopeptides in treated groups were compared with the control group after normalizing VP30 expression using global normalization in SIEVE 2.1. Phosphopeptides ^107^KT*c*GSVEQQLNI*t*APKDSR^125^ and ^107^KT*c*GSVEQQLNITAPKD*s*R^125^ were identified with high confidence (X_Corr_ = 2.71) (Figure 3B) suggesting phosphorylation of Thr-119 and Ser-124 residues. These two phosphopeptides were present at higher levels in 1E7-07 treated cells (3.4-fold increase) and okadaic acid -treated cells (3.3-fold increase) (Figure 3A). Thus, 1E7-07 significantly induced VP30 phosphorylation at Thr-119 and Ser-124 similar to the effect of okadaic acid.

### Identification of Potential PP1 Binding Sites for 1E7-07 by Protein Painting and Molecular Docking

**FIGURE 4:**
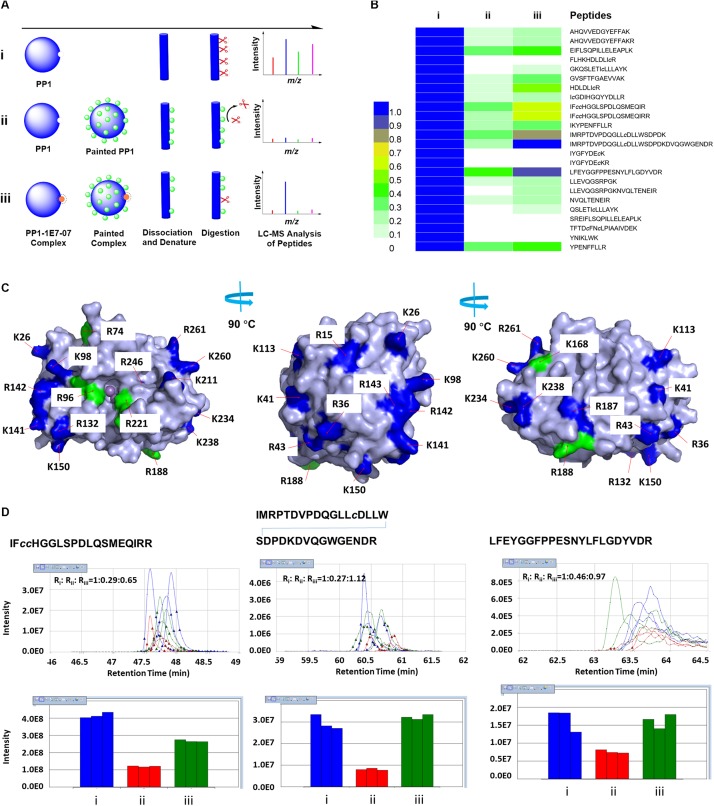
Identification of PP1 binding sites for 1E7-07 by protein painting. **(A)** Schematic of the protein painting workflow showing (i) unpainted PP1; (ii) PP1 painted by molecule ELNLLB; and (iii) protection of PP1 from ELNLLB painting by 1E7-07. **(B)** Heat map of peptides derived from PP1 quantified among groups (i), (ii), and (iii). Label-free quantification using SIEVE 2.1 software with identified PP1 peptides as framing seeds. The scale from 0-1 represents the ratio of group (ii) or (iii) vs. group (i). **(C)** 1E7-07 binding protected PP1 from ELNLLB painting. All cleavage sites except K111 and R122 were mapped on the surface of PP1 and colored blue or green, among which the major protection sites of 1E7-07 from ELNLLB painting were colored green. The models were generated with PyMOL 1.5. **(D)** SIEVE quantitative data of major protection peptides. Ion elution profiles (top) and integrated intensities (bottom) are shown in blue for (i), in red for (ii), and in green for (iii). The data was shown in triplicate and the ratios among different groups were also labeled.

We previously showed that 1E7-03 competes for PP1 binding with an RVxF- containing pRb-Tat peptide. This peptide contains a phosphopeptide derived from Rb protein fused to HIV-1 Tat RVxF containing peptide (Lin et al., 2017). Based on SPR analysis, 1E7-03 bound to more than one site on PP1 whereas C31 bound primarily to the PP1 C-terminal groove (Ammosova et al., 2018). To extend these previous studies, we physically mapped the 1E7-07 binding sites on PP1 with the use of a modified “protein painting” procedure for mapping of interacting protein interfaces (Luchini et al., 2014). We modified the original procedure by applying a covalent paint to lysine and arginine residues, EZ-Link NHS-LC-LC-Biotin (ELNLLB) as opposed to the previously reported labile affinity dyes (see workflow in Figure 4A). We expected non-painted PP1 to be fully accessible for trypsin digestion followed by LC-MS detection of the peptides (Figure 4A, group i). In the presence of ELNLLB paint, surface exposed lysine and arginine residues are blocked and protected from trypsin digestion (Figure 4A, group ii). Pre-treatment with 1E7-07 will compete with ELNLLB paint within the compound binding site and allow trypsin digestion and detection of the peptides within the binding site (Figure 4A, group iii). In the control group i, the majority of PP1-derived peptides were detected (73% coverage, 24 peptides) (Figure 4B). These twenty-four PP1-derived peptides were quantified between groups i-iii using SIEVE 2.1 (Figure 4B). ELNLLB painting blocked most of the twenty-four peptides preventing PP1 from trypsin cleavage by at least 50% (Figure 4B, group ii). All lysine and arginine residues of PP1, with the exception of residues Lys-111 and Arg-122, were located on the PP1 surface supporting the observation of global painting (Figure 4C, labeled with blue and green color). Pretreatment with 1E7-07 protected several residues from ELNLLB painting including Arg-74, Arg-96, Lys-168, Arg-188, and Arg-221 by recovering peptide signals over 70% compared to the control (Figure 4B, group iii and Figure 4C, labeled with green color). The MS spectra from 1E7-07-protected peptides showed strong signal increase in the 1E7-07 treated samples (group iii) compared to the painted samples without treatment (group ii) (Figure 4D). Most prominently protected peptides included ^75^LFEYGGFPPESNYLFLGDYVDR^96^, ^169^IF*cc* HGGLSPDLQSMEQIRR^188^ and ^189^IMRPTDVPDQGLL*c*DLLW SDPDKDVQGWGENDR^221^ (Figure 4D), suggesting that residues Arg-74, Arg-96, Lys-168, Arg-188, and Arg-221 could form a pocket in which 1E7-07 binds (Figure 4C, labeled with green color). In contrast, peptides derived from the unprotected sites showed little or no change in ion current intensity (Supplementary Figure S1).

**FIGURE 5:**
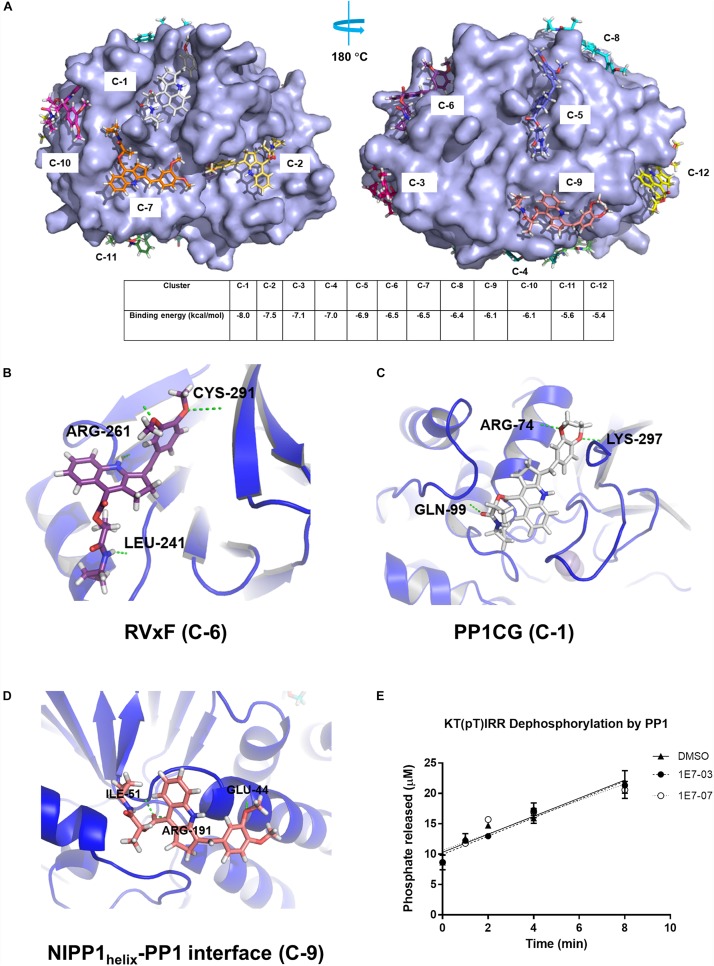
Identification of PP1 binding sites for 1E7-07 by molecular docking. Docking simulations using the Blind Docking Web Server as described in Section “Materials and Methods.” **(A)** A total of 283 binding positions were generated and sorted into 12 clusters according to the binding energy (from –5.4 to –8.0 kcal/mol). **(B)** 1E7-07 binds to the RVxF site of PP1 and forms hydrogen bonds with Leu-241, Arg-261 and Cys-291 (–6.5 kcal/mol, position C-6). **(C)** 1E7-07 binds to the PP1 C-terminal groove and forms hydrogen bonds with Arg-74, Gln-99, and Lys-297 (–8.0 kcal/mol, position C-1). **(D)** 1E7-07 binds to the NIPP1_helix_-PP1 interface with hydrogen bond formation with residues Glu-44, Ile-55, and Arg-191 (–6.1 kcal/mol, position C-9). The residues in close proximity to the ligand (cut-off distance of 8 Å) determined by PyMOL 1.5 software. **(E)** Effect of 1E7-07 and 1E7-03 on PP1α catalytic activity was measured using malachite green dephosphorylation assay (as described in section Materials and Methods) with PP1α and KT(pT)IRR peptide as substrate. The reactions were carried for the indicated time in the presence of 100 μM 1E7-07, 100 μM 1E7-03 or DMSO as vehicle control. Released phosphate concentration was determined by the absorbance of malachite green and a calibration curve.

We used blind docking web-based server^2^ to map 1E7-07 potential binding sites on PP1 *in silico* and align them with the identified protected residues. In total, 283 binding configurations were generated and grouped into 12 clusters based on their position. These were further sorted based on binding energy (range from −5.4 to −8.0 kcal/mol) (Figure 5A). 1E7-07 bound to the RVxF site of PP1 with an energy of −6.5 kcal/mol (cluster C-6, Figure 5A) and formed hydrogen bonds with residues Leu-241, Arg-261 and Cys-291 (Figure 5B). Thus, 1E7-07 binding to the RVxF site might protect Lys-168 from painting as observed above (Figure 4C). 1E7-07 also binds to the PP1 C-terminal groove (PP1CG) with the lowest energy at −8.0 kcal/mol (Figure 5A). Within this binding site, 1E7-07 forms hydrogen bonds with Arg-74, Gln-99, and Lys-297 (Figure 5C). These residues are present in peptide ^75^LFEYGGFPPESNYLFLGDYVDR^96^ which is protected by over 90% from the ELNLLB painting (Figures 4B,D), suggesting that PP1CG is another potential binding site for 1E7-07. In addition, 1E7-07 interacts with the NIPP1_helix_ -binding site (NIPP1_helix_ -PP1 interface) with an energy of -6.1 kcal/mol (Figure 5A, C-9). This binding can protect Arg-188 from ELNLLB painting (Figure 4C) suggesting that 1E7-07 might also bind to the NIPP1_helix_-PP1 interface and forms hydrogen bonds with Glu-44 and Ile-51 (Figure 5D). Although 1E7-07 also protected Arg-96 and Arg-221 residues, which are close to the catalytic site of PP1, 1E7-07 had no effect on PP1 enzymatic activity (Figure 5E) and, therefore, is not likely to target the catalytic site of PP1. Taken together, painting and docking data suggest that 1E7-07 may bind to the RVxF site, the PP1CG site and NIPP1_helix_-PP1 interface.

### 1E7-07 Binds Primarily to the RVxF Site on PP1

**FIGURE 6:**
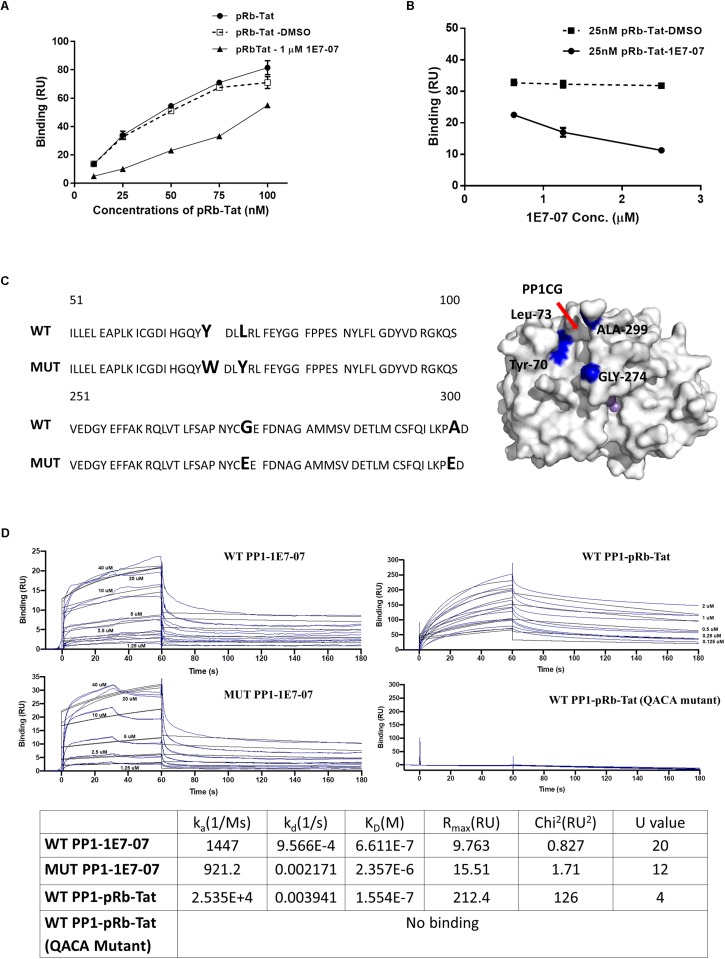
Validation of 1E7-07’s major binding sites on PP1 by SPR. Binding of 1E7-07 to recombinant wild type (WT) or Mutated (MUT) PP1 measured by surface plasmon resonance (SPR). **(A)** Binding competition assay with pRb-Tat peptide for binding to PP1. Different concentrations of pRb-Tat hybrid peptide (10–100 nM) were prepared in a running buffer that contained 1 μM 1E7-07 or DMSO. **(B)** The experiment in **(A)** was repeated by adding different concentrations (0.625–2.5 μM) of 1E7-07 to the running buffer and 25 nM of pRb-Tat peptide binding to PP1 was measured. **(C)** PP1 was mutated in the C-terminal groove to generate Y73W, L73Y, G274E, and A299E mutant. The sequence alignment of WT PP1 and MUT PP1 is shown. The mutation residues on the PP1CG site are also mapped on the surface of PP1 in blue (right panel). **(D)** Binding affinities of 1E7-07 interaction with WT PP1 and MUT PP1 were measured. pRb-Tat and pRb-Tat with QACA mutation were used as a positive and negative control, respectively. *X* axis represents time in seconds and *Y* axis shows binding in resonance units. Each line represents a different concentration of 1E7-07 (0–40 μM). Colored lines are the actual data; black lines are the curve fit that was used to calculate kinetics values. Each concentration was run two times. The binding parameters including k_a_, k_D_, and K_D_ are shown.

To determine which PP1 binding site is targeted by 1E7-07, a competition assay was conducted with pRb-Tat peptide previously shown to bind the RVxF site (Ammosova et al., 2012; Lin et al., 2017). Surface Plasmon Resonance was used to analyze PP1 binding of 1E7-07. Recombinant PP1 was immobilized on CM5 chips by amine coupling. PP1 binding to pRb-Tat was tested in the presence and absence of 1E7-07. For the competition experiment, 1E7-07 (1 μM) or the vehicle control (DMSO) was added to the running buffer. This test resulted in competition of 1E7-07 with pRb-Tat peptide binding to PP1 (Figure 6A), while DMSO had no effect on pRb-Tat binding (Figure 6A). Similarly, 1E7-07 competed dose-dependently when added at 0.625 μM – 2.5 μM concentrations with pRb-Tat peptide added at a constant concentration of 25 nM (Figure 6B). This result further supports the idea that 1E7-07 binds to PP1 through the RVxF motif similarly to 1E7-03, which competes with the pRb-Tat peptide (Lin et al., 2017). To determine whether 1E7-07 binds directly to the PP1CG site, PP1 mutants Y70W, L73Y, G274E and A299E were generated (Figure 6C). This mutant (MUT) PP1 maintained enzymatic activity similar to wild type (WT) PP1 indicating that mutations within the C-terminal groove of PP1 had no effect on PP1 enzymatic activity and did not change the catalytic site of PP1 (Ammosova et al., 2018). We compared 1E7-07 binding affinity to WT and MUT PP1 (Figure 6D). The pRb-Tat peptide was used as a positive control and bound with K_D_ = 1.554 × 10^–7^ M (Figure 6D). The QACA mutant pRb-Tat did not bind to PP1 and was used as a negative control (Figure 6D). While 1E7-07 showed strong binding to WT PP1 (K_D_ = 6.611 × 10^–7^ M, Figure 6D), it showed a moderate 3-fold reduction for mutant PP1 (K_D_ = 2.357 × 10^–6^ M, Figure 6D), suggesting that PP1CG is not the major binding site.

**FIGURE 7:**
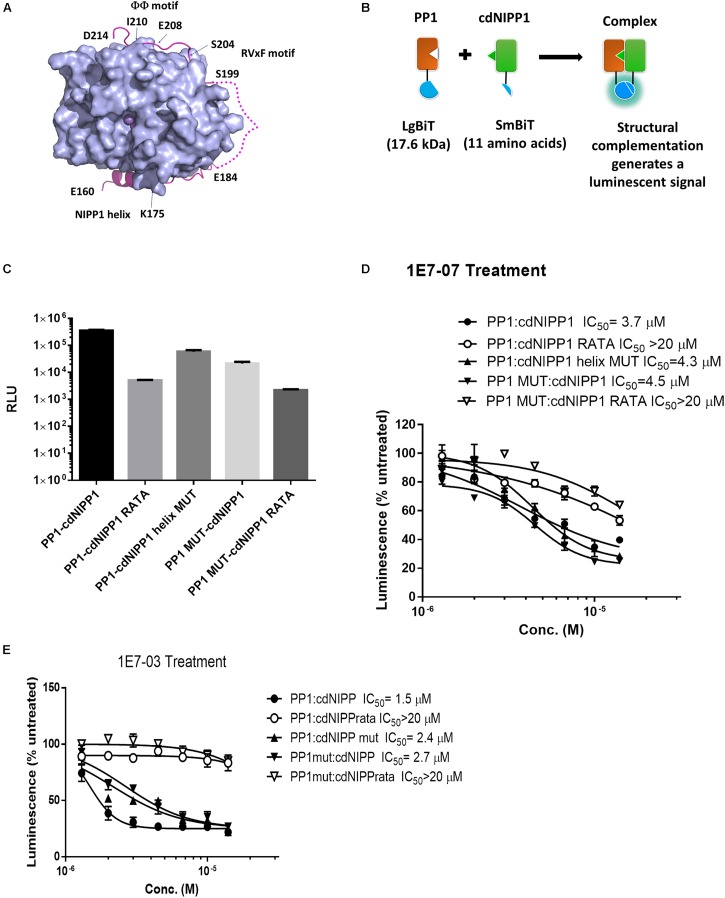
Validation of 1E7-07’s major binding sites on PP1 by NanoBiT assay. **(A)** The PP1-NIPP1 interaction. Cartoon representation of PP1α_7__–__300_ (light blue surface) and NIPP1_160__–__214_ (light magenta) complex. The Mn^2+^ ions at active site of PP1 represented as purple spheres. Three major NIPP1 interaction motifs including RVxF motif (199–204), NIPP1 helix (160–175) and ΦΦ motif (208–210) were labeled. NIPP1 residues 185–198 were not observed in crystal complex (PDB: 3V4Y) and displayed as a light magenta dashed line. **(B)** Schematic representation of PP1:cdNIPP measured by NanoBiT complementation assay. PP1/PP1MUT was fused with LgBiT- at the C-terminus, while cdNIPP1/cdNIPP1 RATA/cdNIPP1 helix MUT was tagged with SmBit- at the C-terminus. Their interactions reconstitute an active NanoLuc and produce luminescence in the presence of a substrate, furimazine. **(C)** LgBiT- and SmBit-tagged genes were co-transfected into HEK293T cells. NanoBit luciferase activity was measured at 24 h post transfection. Each value represents the mean ± SD from three independent cultures. **(D,E)** After co-transfection of NanoBiT-tagged genes for 24 h, cells were treated with different concentrations (1.3–14 μM) of 1E7-07 or 1E7-03 for additional 6 h and NanoBiT luciferase activity was measured. Data are the mean ± SD of triplicates.

To further delineate the 1E7-07 binding site on PP1, a novel split NanoBiT assay (Dixon et al., 2016) was developed to analyze the effect of 1E7-07 on the interaction of PP1 with a well characterized central domain of nuclear inhibitor of PP1 (cdNIPP1, residues 140-225). NIPP1 interacts with PP1 through three distinct sites including the RVxF motif ^199^SRVTFS^204^, NIPP1 helix ^160^ETELDNLTEFNTAHNK^175^ and ΦΦ motif ^208^EII^210^ (O’Connell et al., 2012) (Figure 7A). We utilized the split NanoBit assay to measure the interaction of PP1, which was fused to the C-terminus of the large bit with the cdNIPP1 that was fused to the C-terminus of the small bit (Figure 7B). To assay the effect of 1E7-07 on PP1-cdNIPP1 interaction, we mutated cdNIPP1 on the RVxF motif (cdNIPP1 RATA, V201A/F203A mutation) and on the helix motif (cdNIPP1 helix mut, D164A/T171A/N174A/K175A/I177A mutations). We also mutated PP1 on the C-terminal groove (PP1 mut, Y70W/L73Y/G274E/A299E mutations). As expected, the RVxF mutation strongly reduced binding of cdNIPP1 to PP1 (70-fold reduction, Figure 7C). In contrast, the cdNIPP1 helix mutation showed moderate reduction in PP1 binding (6-fold, Figure 7C). Mutation of the C-terminal groove of PP1 also reduced cdNIPP1 binding (17-fold, Figure 7C). Mutations of the PP1 C-terminal groove and the cdNIPP1 RVxF motif showed the strongest reduction in binding (160-fold, Figure 7C). These results suggest that the RVxF motif is the strongest contributor to the PP1:cdNIPP1 interaction. To test the effect of 1E7-07, HEK293T cells co-transfected for 24 h with the indicated NanoBiT plasmids were treated with different concentrations (1.3–14 μM) of 1E7-07 for 6 h (Figure 7D). 1E7-07 competes with PP1:cdNIPP1 binding with IC_50_ = 3.7 μM. It also competes similarly with PP1MUT:cdNIPP1 binding (IC_50_ = 4.5 μM), suggesting that PP1CG does not contribute significantly to the interaction of 1E7-07 with PP1 (Figure 7D). Correspondingly, 1E7-07 also competes similarly with PP1:cdNIPP1 MUT binding (IC_50_ = 4.3 μM), suggesting that the PP1:NIPP1 helix interface is not likely to serve as a binding site for 1E7-07 (Figure 7D). In contrast, 1E7-07 had no effect on PP1:cdNIPP1 RATA and PP1 MUT:cdNIPP1 RATA interactions (IC_50_ > 20 μM), further suggesting that RVxF is the main binding site for 1E7-07 (Figure 7E). This observation supports the above competition of 1E7-07 with the pRb-Tat peptide binding to PP1. A slight increase in IC_50_s for 1E7-07 competition with PP1:cdNIPP1RATA and PP1 mut:cdNIPP1 RATA interactions suggest that 1E7-07 might also have weak interactions with PP1CG and the NIPP1 helix binding site. This indication is also in line with the observed protection of Arg-74 and Arg-188 residues observed in the painting experiment. Moreover, we observed similar effect of 1E7-03 on PP1:cdNIPP1 interaction in NanoBiT assays (Figure 7C). We observed lower IC_50_s for 1E7-03 which could explain its slightly better EBOV inhibitory activities. Taken together, these results suggest that 1E7-07 and 1E7-03 bind to multiple sites of PP1 with the RVxF being the major binding site.

## Discussion

The optimization of ‘drug-like’ properties of new chemical entities has always played a critical role in the drug discovery process. Here, we identified a new EBOV inhibitory compound, 1E7-07, that targets the PP1 RVxF binding site. 1E7-07 showed significantly improved metabolic stability and similar EBOV inhibitory activity compared with the original compound 1E7-03. EBOV VP30 is a viral transcriptional regulator that interacts with NP, VP35 and L. VP30 phosphorylation functions as a switch between EBOV transcription and replication (Ilinykh et al., 2014). We showed that expression of cdNIPP1 promotes VP30 phosphorylation suggesting the role of PP1 in VP30 dephosphorylation (Ilinykh et al., 2014). NP binds PP2A which dephosphorylates VP30 and promotes viral transcription (Kruse et al., 2018). VP30 might also bind additional host factors including ubiquitin ligase RBBP6 that mimics NP and disrupts VP30-NP interaction (Batra et al., 2018). Here we used label-free quantitative mass spectrometry to show that 1E7-07 significantly upregulates phosphorylation of VP30 residues Thr-119 and Ser-124. We recently showed that the PP1-targeting compound C31 also induces Thr-119 and Ser-124. Further analysis is necessary to understand the role these residues play in phosphorylation and EBOV transcription regulation.

To identify the PP1 binding site of 1E7-07, we developed a modified “protein painting” approach. This approach combined with molecular docking analysis, determined the RVxF site, PP1CG and NIPP1 helix binding interface to be potential binding sites of 1E7-07. The employment of ELNLLB as a painting molecule that forms covalent bonds reduces false positives which can be observed upon the dissociation of the painting molecule during protein digestion (Dailing et al., 2015). Combining protein painting with docking simulation facilitated further refinement and assignment of the potential binding sites. Overall, this method is rapid and requires no special software beyond a label-free proteomics workflow. Two limitations of protein painting are its dependence on the presence of trypsin cleavage sites on the surface of the protein under investigation, and the density of the sites, which both can limit detection of the binding sites. Substantial coverage is also required to detect and identify small molecule binding sites. While we were able to use the painting approach for 1E7-07, it did not work for okadaic acid. This could be due to the flexibility of okadaic acid and/or poor competition with the painting molecule.

We first validated binding sites with SPR which showed that 1E7-07 bound primarily to the RVxF site of PP1 and less to the PP1CG site. We followed SPR validation with a novel split NanoBiT system that detected the interaction of PP1 with cdNIPP1 in cultured cells. We showed that 1E7-07 primarily competes for the RVxF binding site when we used combinations of PP1 and cdNIPP1 with mutations in the PP1 C-terminal groove, cdNIPP1 RVxF and helix motifs. Previous structural analysis shows that NIPP1 residues Val-201 and Phe-203 are inserted into a hydrophobic pocket formed by PP1 residues Ile-169, Leu-243, Phe-257, Arg-261, Val-264, Leu-266, Met-283, Leu-289, Cys-291, and Phe-293 (O’Connell et al., 2012). The binding is further enhanced by hydrogen bonds between NIPP1 Val-201: PP1 Asp-242 and NIPP1 Thr-202: PP1 Met-283/Cys-291. Our docking data shows that the ethenylbenzene moiety of 1E7-07 fits in the same hydrophobic pocket allowing for a hydrogen bond formation between one of the methoxy groups of 1E7-07 and PP1 Cys-291. This might explain why the *meta*- and *para*- substitution of the methoxy group on the ethenylbenzene moiety was important for maintaining EBOV inhibitory activity. We showed previously that co-expression of cdNIPP1 and VP30 increased VP30 phosphorylation (Ilinykh et al., 2014), likely by sequestering PP1 in the nucleus and reducing the availability of PP1 in the cytoplasm (see Supplementary Figure S2). As 1E7-07 does not affect PP1 cellular localization (Supplementary Figure S2), it is likely to induce VP30 phosphorylation by disrupting the interaction of PP1 with a yet identified cytoplasmic regulatory subunit of PP1 that recruits PP1 to the EBOV transcription complex. Taken together, our findings indicate that 1E7-07 inhibits EBOV at least in part by targeting the RVxF site of PP1.

## Data Availability

The raw data supporting the conclusions of this manuscript will be made available by the authors, without undue reservation, to any qualified researcher.

## Author Contributions

XL, TA, CP, AI, AA, YS, and NK conducted the experiments. MC provided recombinant PP1 for the painting analysis. DK designed small molecules. AÜ, WP, AB, and SN designed the experiments and wrote the manuscript. SN conceptualized the study and provided the overall supervision.

## Conflict of Interest Statement

The authors declare that the research was conducted in the absence of any commercial or financial relationships that could be construed as a potential conflict of interest.

## Footnotes

1 http://bio-hpc.ucam.edu/webBD/index.php/entry

2 http://bio-hpc.ucam.edu/webBD/index.php/entry
