# Supplementary material for: Targeting the Non-catalytic RVxF Site of Protein Phosphatase-1 With Small Molecules for Ebola Virus Inhibition
Source: Front Microbiol. 2019 Sep 13;10:2145. doi: 10.3389/fmicb.2019.02145 (PMC6753193; doi:10.3389/fmicb.2019.02145)

Supplemental Figure S1: Peptides of PP1 that were not protected by 1E7-07 from ELNLLB painting. Ion elution profiles (top) and integrated intensities (bottom) of control (group i), ELNLLB painting (group ii) and PP1-1E7-07 complex with ELNLLB painting (group iii) are shown in blue, red and green, respectively.

Supplemental Figure S2: 1E7-07 does not affect cellular localization of PP1. (A) 293T cells were transfected with PP1-mCherry vector and treated with 1E7-07 for 24 hr. At twenty-four hr post transfection, the cells were treated with 10 µM 1E7-07 or DMSO as vehicle control. Cells also stained with Hechts. At fourty-eight hr posttransfection the cells were photographed on Olympus IX73 using filters for Texas Red and Hechts fluorescence at 600X magnification. (B) PP1 is translocated to the nucleus when co-expressed with cdNIPP1. 293T cells were transfected with vectors expressing PP1γ-mCherry or PP1γ-mCherry and cdNIPP1-EGFP. At forty-eight hr posttransfection the cells were photographed on Olympus IX73 using filters for Texas Red and FITC fluorescence with 400X magnification.

*Corresponding author contact information: snekhai@howard.edu, Phone +12022388396

Supplemental Figure S1

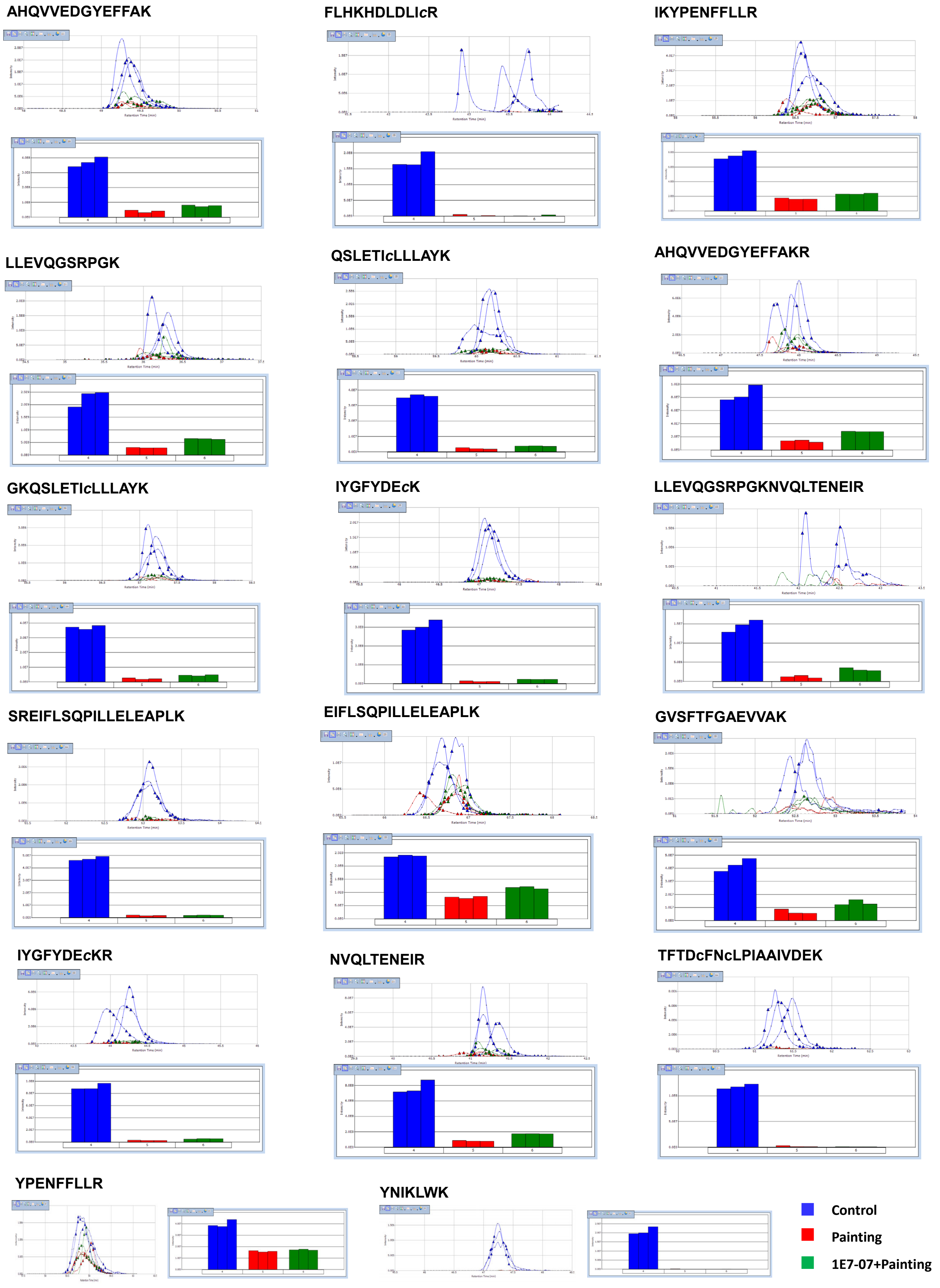

Supplemental Figure S2

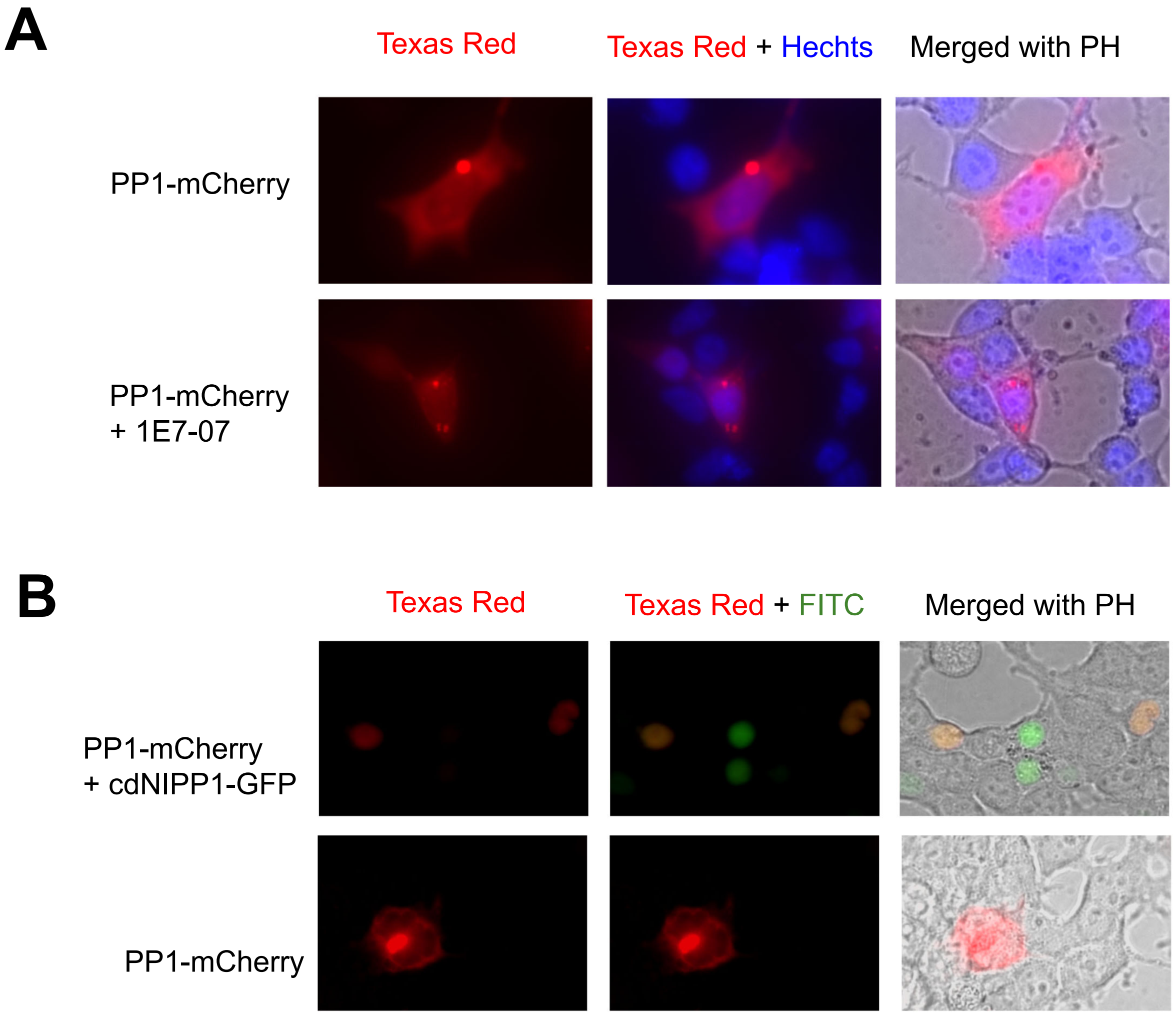

Supplement: Supplementary file 1 [file Data_Sheet_1.pdf]
